# Supplementary material for: Novel Universal Recombinant Rotavirus A Vaccine Candidate: Evaluation of Immunological Properties
Source: Viruses. 2024 Mar 12;16(3):438. doi: 10.3390/v16030438 (PMC10976063; doi:10.3390/v16030438)
Supplement: Supplementary file 1 [file viruses-16-00438-s001.zip › Table S1 .pdf]

Antigen	Antigen concentration, *μg/ml*	A_{450} value		
		Replicate 1	Replicate 2	Geometric mean
**URRA**	10	1.78	1.72	1.74
	50	1.84	1.8	1.82
	100	1.88	1.73	1.80
	200	1.97	1.88	1.93
**SPs**	10	0.21	0.21	0.21
	50	0.53	0.52	0.52
	100	0.36	0.35	0.36
	200	0.18	0.17	0.17
