# Supplementary material for: Novel Universal Recombinant Rotavirus A Vaccine Candidate: Evaluation of Immunological Properties
Source: Viruses. 2024 Mar 12;16(3):438. doi: 10.3390/v16030438 (PMC10976063; doi:10.3390/v16030438)
Supplement: Supplementary file 1 [file viruses-16-00438-s001.zip › Table S2.pdf]

Antigen	Antigen concentration, *μg/ml*	A_{450} value		
		Replicate 1	Replicate 2	Geometric mean
**URRA**	10	1.27	1.32	1.30
	50	1.67	1.7	1.68
	100	1.67	1.68	1.67
	200	1.57	1.71	1.64
**SPs**	10	0.20	0.18	0.19
	50	0.65	0.63	0.64
	100	0.38	0.38	0.38
	200	0.15	0.16	0.16
