# Supplementary material for: Novel Universal Recombinant Rotavirus A Vaccine Candidate: Evaluation of Immunological Properties
Source: Viruses. 2024 Mar 12;16(3):438. doi: 10.3390/v16030438 (PMC10976063; doi:10.3390/v16030438)
Supplement: Supplementary file 1 [file viruses-16-00438-s001.zip › Table S3.pdf]

IgG to URRA							
**Immunisation group**	**Identification number of mouse**	**Titre**	**log_{10}(titre)**	**Immunisation group**	**Identification number of mouse**	**Titre**	**log_{10}(titre)**
**Group 1 (Non-immunised)**	1.1	7.36×10^1	1.87	**Group 3 (URRA)**	3.1	1.57×10^2	2.2
	1.2	3.36×10^2	2.53		3.2	3.88×10^1	1.59
	1.3	6.56×10^1	1.82		3.3	6.47×10^2	2.81
	1.4	7.23×10^1	1.86		3.4	6.72×10^1	1.83
	1.5	2.25×10^2	2.35		3.5	3.0×10^1	1.48
	1.6	3.0×10^1	1.48		3.6	3.0×10^1	1.48
	1.7	3.0×10^1	1.48		3.7	2.33×10^3	3.37
	1.8	6.65×10^1	1.82		3.8	1.95×10^2	2.29
	1.9	3.0×10^1	1.48		3.9	1.6×10^2	2.2
	1.10	7.92×10^1	1.9		3.10	2.33×10^2	2.37
	Median	6.94×10^1	1.84		Median	1.59×10^2	2.2
**Group 2 (SPs)**	2.1	5.99×10^1	1.78	**Group 4 (URRA + SPs)**	4.1	7.06×10^2	2.85
	2.2	5.34×10^1	1.73		4.2	6.31×10^2	2.8
	2.3	1.98×10^2	2.3		4.3	2.23×10^2	2.35
	2.4	7.64×10^1	1.88		4.4	2.68×10^2	2.43
	2.5	7.14×10^1	1.85		4.5	2.12×10^2	2.33
	2.6	3.0×10^1	1.48		4.6	1.9×10^3	3.28
	2.7	7.69×10^1	1.89		4.7	7.78×10^2	2.89
	2.8	1.84×10^2	2.26		4.8	1.96×10^2	2.29
	2.9	5.97×10^1	1.78		4.9	2.24×10^2	2.35
	2.10	6.32×10^1	1.8		4.10	6.39×10^2	2.81
	Median	6.73×10^1	1.83		Median	4.5×10^2	2.61
