# Supplementary material for: Novel Universal Recombinant Rotavirus A Vaccine Candidate: Evaluation of Immunological Properties
Source: Viruses. 2024 Mar 12;16(3):438. doi: 10.3390/v16030438 (PMC10976063; doi:10.3390/v16030438)
Supplement: Supplementary file 1 [file viruses-16-00438-s001.zip › Table S4.pdf]

IgG to URRA							
**Immunisation group**	**Identification number of mouse**	**Titre**	**log_{10}(titre)**	**Immunisation group**	**Identification number of mouse**	**Titre**	**log_{10}(titre)**
**Group 1 (Non-immunised)**	1.11	2.49×10^2	2.4	**Group 3 (URRA)**	3.11	2.23×10^3	3.35
	1.12	1.47×10^3	3.17		3.12	6.69×10^3	3.83
	1.13	2.05×10^2	2.31		3.13	1.99×10^3	3.30
	1.14	4.8×10^2	2.68		3.14	5.02×10^3	3.7
	1.15	1.47×10^2	2.17		3.15	1.21×10^3	3.08
	1.16	8.62×10^1	1.94		3.16	2.16×10^3	3.33
	1.17	8.82×10^1	1.95		3.17	5.29×10^3	3.72
	1.18	8.02×10^1	1.9		3.18	5.43×10^2	2.73
	1.19	7.52×10^1	1.88		3.19	4.58×10^3	3.66
	1.20	8.07×10^1	1.91		3.20	4.04×10^3	3.61
	1.21	3.29×10^2	2.52		3.21	1.33×10^3	3.12
	1.22	2.67×10^2	2.43		3.22	1.19×10^3	3.08
	1.23	2.18×10^2	2.34		3.23	1.03×10^3	3.01
	1.24	2.61×10^2	2.42		3.24	2.46×10^2	2.39
	1.25	3.0×10^1	1.48		3.25	6.69×10^2	2.83
	Median	2.05×10^2	2.31		Median	1.99×10^3	3.3
**Group 2 (SPs)**	2.11	2.54×10^2	2.4	**Group 4 (URRA + SPs)**	4.11	3.38×10^4	4.53
	2.12	1.95×10^2	2.29		4.12	1.0×10^5	5.0
	2.13	2.35×10^2	2.37		4.13	1.41×10^4	4.15
	2.14	2.52×10^2	2.4		4.14	3.0×10^4	4.48
	2.15	2.62×10^2	2.42		4.15	2.71×10^3	3.43
	2.16	8.3×10^1	1.92		4.16	9.07×10^3	3.96
	2.17	5.47×10^1	1.74		4.17	1.12×10^3	3.05
	2.18	8.97×10^1	1.95		4.18	5.67×10^4	4.75
	2.19	6.75×10^1	1.83		4.19	2.18×10^4	4.34
	2.20	2.34×10^2	2.37				
	2.21	2.03×10^2	2.31				
	2.22	3.97×10^2	2.6				
	2.23	2.49×10^2	2.4				
	2.24	1.31×10^2	2.12				
	2.25	8.76×10^1	1.94				
	Median	2.03×10^2	2.31		Median	2.18×10^4	4.34
