# Supplementary material for: Novel Universal Recombinant Rotavirus A Vaccine Candidate: Evaluation of Immunological Properties
Source: Viruses. 2024 Mar 12;16(3):438. doi: 10.3390/v16030438 (PMC10976063; doi:10.3390/v16030438)
Supplement: Supplementary file 1 [file viruses-16-00438-s001.zip › Table S5.pdf]

IgG1 to URRA							
**Immunisation group**	**Identification number of mouse**	**Titre**	**log_{10}(titre)**	**Immunisation group**	**Identification number of mouse**	**Titre**	**log_{10}(titre)**
**Group 1 (Non-immunised)**	1.11	5.06×10^1	1.7	**Group 3 (URRA)**	3.11	5.33×10^2	2.73
	1.12	1.02×10^2	2.01		3.12	1.92×10^3	3.28
	1.13	5.25×10^2	2.72		3.13	1.42×10^3	3.15
	1.14	8.65×10^1	1.94		3.14	6.78×10^2	2.83
	1.15	8.41×10^1	1.92		3.15	1.13×10^3	3.05
	1.16	7.4×10^1	1.87		3.16	2.27×10^3	3.36
	1.17	8.25×10^1	1.92		3.17	1.18×10^3	3.07
	1.18	5.29×10^1	1.72		3.18	6.05×10^2	2.78
	1.19	1.31×10^2	2.12		3.19	2.9×10^3	3.46
	1.20	3.0×10^1	1.48		3.20	2.08×10^3	3.32
	1.21	8.01×10^1	1.9		3.21	1.53×10^3	3.18
	1.22	2.48×10^3	3.39		3.22	2.39×10^3	3.38
	1.23	2.66×10^2	2.43		3.23	2.22×10^2	2.35
	1.24	1.63×10^2	2.21		3.24	1.5×10^3	3.18
	1.25	2.11×10^2	2.32		3.25	2.33×10^3	3.37
	Median	8.65×10^1	1.94		Median	1.5×10^3	3.18
**Group 2 (SPs)**	2.11	8.26×10^1	1.92	**Group 4 (URRA + SPs)**	4.11	3.38×10^4	4.53
	2.12	5.76×10^2	2.76		4.12	4.51×10^4	4.65
	2.13	5.85×10^2	2.77		4.13	1.17×10^5	5.07
	2.14	1.13×10^2	2.05		4.14	1.89×10^5	5.28
	2.15	1.46×10^2	2.16		4.15	1.75×10^4	4.24
	2.16	3.0×10^1	1.48		4.16	2.38×10^3	3.38
	2.17	3.0×10^1	1.48		4.17	1.3×10^3	3.11
	2.18	8.2×10^1	1.91		4.18	5.93×10^5	5.77
	2.19	1.16×10^2	2.06		4.19	5.45×10^4	4.74
	2.20	1.91×10^2	2.28				
	2.21	2.81×10^2	2.45				
	2.22	4.34×10^2	2.64				
	2.23	2.23×10^2	2.35				
	2.24	9.98×10^2	3.0				
	2.25	7.36×10^1	1.87				
	Median	1.46×10^2	2.16		Median	4.51×10^4	4.65
