# Supplementary material for: Novel Universal Recombinant Rotavirus A Vaccine Candidate: Evaluation of Immunological Properties
Source: Viruses. 2024 Mar 12;16(3):438. doi: 10.3390/v16030438 (PMC10976063; doi:10.3390/v16030438)
Supplement: Supplementary file 1 [file viruses-16-00438-s001.zip › Table S6.pdf]

IgG2a to URRA							
Immunisation group	Identification number of mouse	Titre	log_{10}(titre)	Immunisation group	Identification number of mouse	Titre	log_{10}(titre)
**Group 1 (Non-immunised)**	1.11	2.05 x 10^3	3.31	**Group 3 (URRA)**	3.11	3.05 x 10^3	3.48
	1.12	3.39 x 10^3	3.53		3.12	6.48 x 10^4	4.81
	1.13	5.14 x 10^3	3.71		3.13	6.12 x 10^3	3.79
	1.14	1.67 x 10^3	3.22		3.14	3.62 x 10^4	4.56
	1.15	7.73 x 10^2	2.89		3.15	6.0 x 10^3	3.78
	1.16	7.04 x 10^3	3.85		3.16	4.07 x 10^3	3.61
	1.17	7.95 x 10^2	2.9		3.17	1.87 x 10^4	4.27
	1.18	1.92 x 10^4	4.28		3.18	9.95 x 10^3	4.0
	1.19	2.03 x 10^4	4.31		3.19	2.18 x 10^4	4.34
	1.20	5.64 x 10^2	2.75		3.20	2.11 x 10^4	4.32
	1.21	7.59 x 10^2	2.88		3.21	5.23 x 10^3	3.72
	1.22	1.99 x 10^4	4.3		3.22	4.36 x 10^3	3.64
	1.23	1.08 x 10^3	3.04		3.23	7.97 x 10^2	2.9
	1.24	7.29 x 10^2	2.86		3.24	1.15 x 10^4	4.06
	1.25	7.03 x 10^3	3.85		3.25	4.18 x 10^3	3.62
	Median	2.05 x 10^3	3.31		Median	6.12 x 10^3	3.79
**Group 2 (SPs)**	2.11	2.37 x 10^3	3.38	**Group 4 (URRA + SPs)**	4.11	4.01 x 10^2	2.6
	2.12	1.66 x 10^3	3.22		4.12	5.69 x 10^5	5.75
	2.13	5.73 x 10^3	3.76		4.13	1.02 x 10^4	4.01
	2.14	5.89 x 10^2	2.77		4.14	3.41 x 10^3	3.53
	2.15	2.25 x 10^3	3.35		4.15	6.32 x 10^3	3.8
	2.16	3.4 x 10^4	4.53		4.16	4.23 x 10^4	4.63
	2.17	8.38 x 10^3	3.92		4.17	1.4 x 10^4	4.15
	2.18	2.11 x 10^4	4.32		4.18	5.26 x 10^4	4.72
	2.19	2.17 x 10^4	4.34		4.19	6.55 x 10^3	3.82
	2.20	2.07 x 10^4	4.32				
	2.21	2.31 x 10^3	3.36				
	2.22	4.69 x 10^3	3.67				
	2.23	2.44 x 10^3	3.39				
	2.24	3.06 x 10^3	3.48				
	2.25	2.23 x 10^3	3.35				
	Median	3.06 x 10^3	3.48		Median	1.02 x 10^4	4.01
