# Supplementary material for: Novel Universal Recombinant Rotavirus A Vaccine Candidate: Evaluation of Immunological Properties
Source: Viruses. 2024 Mar 12;16(3):438. doi: 10.3390/v16030438 (PMC10976063; doi:10.3390/v16030438)
Supplement: Supplementary file 1 [file viruses-16-00438-s001.zip › Table S7.pdf]

IgG2b to URRA							
**Immunisation group**	**Identification number of mouse**	**Titre**	**log_{10}(titre)**	**Immunisation group**	**Identification number of mouse**	**Titre**	**log_{10}(titre)**
**Group 1 (Non-immunised)**	1.11	1.69×10^4	4.23	**Group 3 (URRA)**	3.11	2.18×10^4	4.34
	1.12	4.76×10^4	4.68		3.12	4.07×10^4	4.61
	1.13	9.07×10^3	3.96		3.13	1.39×10^4	4.14
	1.14	3.28×10^3	3.52		3.14	1.09×10^4	4.04
	1.15	2.12×10^4	4.33		3.15	5.88×10^3	3.77
	1.16	5.93×10^2	2.77		3.16	1.55×10^3	3.19
	1.17	6.51×10^2	2.81		3.17	2.08×10^4	4.32
	1.18	4.38×10^3	3.64		3.18	2.55×10^2	2.41
	1.19	2.79×10^3	3.45		3.19	5.67×10^4	4.75
	1.20	1.17×10^3	3.07		3.20	2.01×10^3	3.3
	1.21	1.91×10^3	3.28		3.21	5.56×10^3	3.75
	1.22	3.13×10^3	3.50		3.22	5.33×10^3	3.73
	1.23	4.5×10^2	2.65		3.23	1.82×10^2	2.26
	1.24	6.61×10^2	2.82		3.24	6.91×10^2	2.84
	1.25	2.44×10^2	2.39		3.25	7.2×10^2	2.86
	Median	2.79×10^3	3.45		Median	5.56×10^3	3.75
**Group 2 (SPs)**	2.11	1.97×10^4	4.29	**Group 4 (URRA + SPs)**	4.11	8.94×10^1	1.95
	2.12	1.16×10^4	4.07		4.12	1.63×10^4	4.21
	2.13	1.76×10^4	4.25		4.13	1.89×10^4	4.28
	2.14	2.28×10^2	2.36		4.14	7.21×10^3	3.86
	2.15	5.32×10^3	3.73		4.15	1.18×10^4	4.07
	2.16	6.2×10^2	2.79		4.16	1.55×10^3	3.76
	2.17	3.0×10^1	1.48		4.17	3.83×10^3	3.58
	2.18	7.33×10^3	3.87		4.18	5.75×10^4	4.76
	2.19	4.13×10^3	3.62		4.19	2.36×10^3	3.37
	2.20	4.57×10^4	4.66				
	2.21	3.68×10^3	3.57				
	2.22	3.28×10^3	3.52				
	2.23	4.23×10^2	2.63				
	2.24	2.61×10^2	2.41				
	2.25	8.01×10^2	2.9				
	Median	3.68×10^3	3.57		Median	7.21×10^3	3.86
