# Supplementary material for: Novel Universal Recombinant Rotavirus A Vaccine Candidate: Evaluation of Immunological Properties
Source: Viruses. 2024 Mar 12;16(3):438. doi: 10.3390/v16030438 (PMC10976063; doi:10.3390/v16030438)
Supplement: Supplementary file 1 [file viruses-16-00438-s001.zip › Table S8.pdf]

IgG3 to URRA							
**Immunisation group**	**Identification number of mouse**	**Titre**	**log_{10}(titre)**	**Immunisation group**	**Identification number of mouse**	**Titre**	**log_{10}(titre)**
**Group 1 (Non-immunised)**	1.11	6.27×10^1	1.8	**Group 3 (URRA)**	3.11	4.2×10^1	1.62
	1.12	3.0×10^1	1.48		3.12	3.0×10^1	1.48
	1.13	3.0×10^1	1.48		3.13	1.77×10^2	2.25
	1.14	1.02×10^2	2.01		3.14	2.2×10^2	2.34
	1.15	8.15×10^1	1.91		3.15	8.2×10^1	1.91
	1.16	3.0×10^1	1.48		3.16	7.48×10^1	1.87
	1.17	2.07×10^2	2.32		3.17	4.58×10^2	2.66
	1.18	3.0×10^1	1.48		3.18	3.0×10^1	1.48
	1.19	3.0×10^1	1.48		3.19	2.53×10^2	2.4
	1.20	9.29×10^2	2.97		3.20	1.93×10^2	2.29
	1.21	6.13×10^2	2.79		3.21	1.32×10^3	3.12
	1.22	2.23×10^2	2.35		3.22	2.19×10^2	2.34
	1.23	2.53×10^2	2.4		3.23	1.35×10^2	2.13
	1.24	2.46×10^2	2.39		3.24	7.3×10^1	1.86
	1.25	8.84×10^1	1.95		3.25	2.02×10^2	2.31
	Median	8.84×10^1	1.95		Median	1.77×10^2	2.25
**Group 2 (SPs)**	2.11	3.0×10^1	1.48	**Group 4 (URRA + SPs)**	4.11	8.23×10^1	1.92
	2.12	3.0×10^1	1.48		4.12	1.51×10^2	2.18
	2.13	3.0×10^1	1.48		4.13	1.69×10^3	3.23
	2.14	5.49×10^1	1.74		4.14	5.26×10^2	2.72
	2.15	7.78×10^1	1.89		4.15	2.79×10^2	2.45
	2.16	8.76×10^1	1.94		4.16	5.79×10^1	1.76
	2.17	4.07×10^1	1.61		4.17	8.66×10^1	1.94
	2.18	3.0×10^1	1.48		4.18	7.62×10^2	2.88
	2.19	3.0×10^1	1.48		4.19	8.45×10^1	1.93
	2.20	7.72×10^2	2.89				
	2.21	2.45×10^2	2.39				
	2.22	2.13×10^2	2.33				
	2.23	1.76×10^2	2.25				
	2.24	6.79×10^1	1.83				
	2.25	3.0×10^1	1.48				
	Median	5.49×10^1	1.74		Median	1.51×10^2	2.18
