# Supplementary material for: Novel Universal Recombinant Rotavirus A Vaccine Candidate: Evaluation of Immunological Properties
Source: Viruses. 2024 Mar 12;16(3):438. doi: 10.3390/v16030438 (PMC10976063; doi:10.3390/v16030438)
Supplement: Supplementary file 1 [file viruses-16-00438-s001.zip › Table S9.pdf]

Identification number of mouse	Total IgG to URRA		Total IgG to SPs	
	Titre	log_{10}(titre)	Titre	log_{10}(titre)
4.11	3.38×10^4	4.53	7.22×10^3	3.86
4.12	1.0×10^5	5.0	5.11×10^3	3.71
4.13	1.41×10^4	4.15	2.02×10^3	3.31
4.14	3.0×10^4	4.48	4.56×10^2	2.66
4.15	2.71×10^3	3.43	1.52×10^3	3.18
4.16	9.07×10^3	3.96	7.94×10^2	2.9
4.17	1.12×10^3	3.05	2.82×10^2	2.45
4.18	5.67×10^4	4.75	1.11×10^4	4.04
4.19	2.18×10^4	4.34	5.77×10^2	2.76
Median	2.18×10^4	4.34	1.52×10^3	3.18
