# Supplementary figures and images for: Novel Universal Recombinant Rotavirus A Vaccine Candidate: Evaluation of Immunological Properties
Source: Viruses. 2024 Mar 12;16(3):438. doi: 10.3390/v16030438 (PMC10976063; doi:10.3390/v16030438)

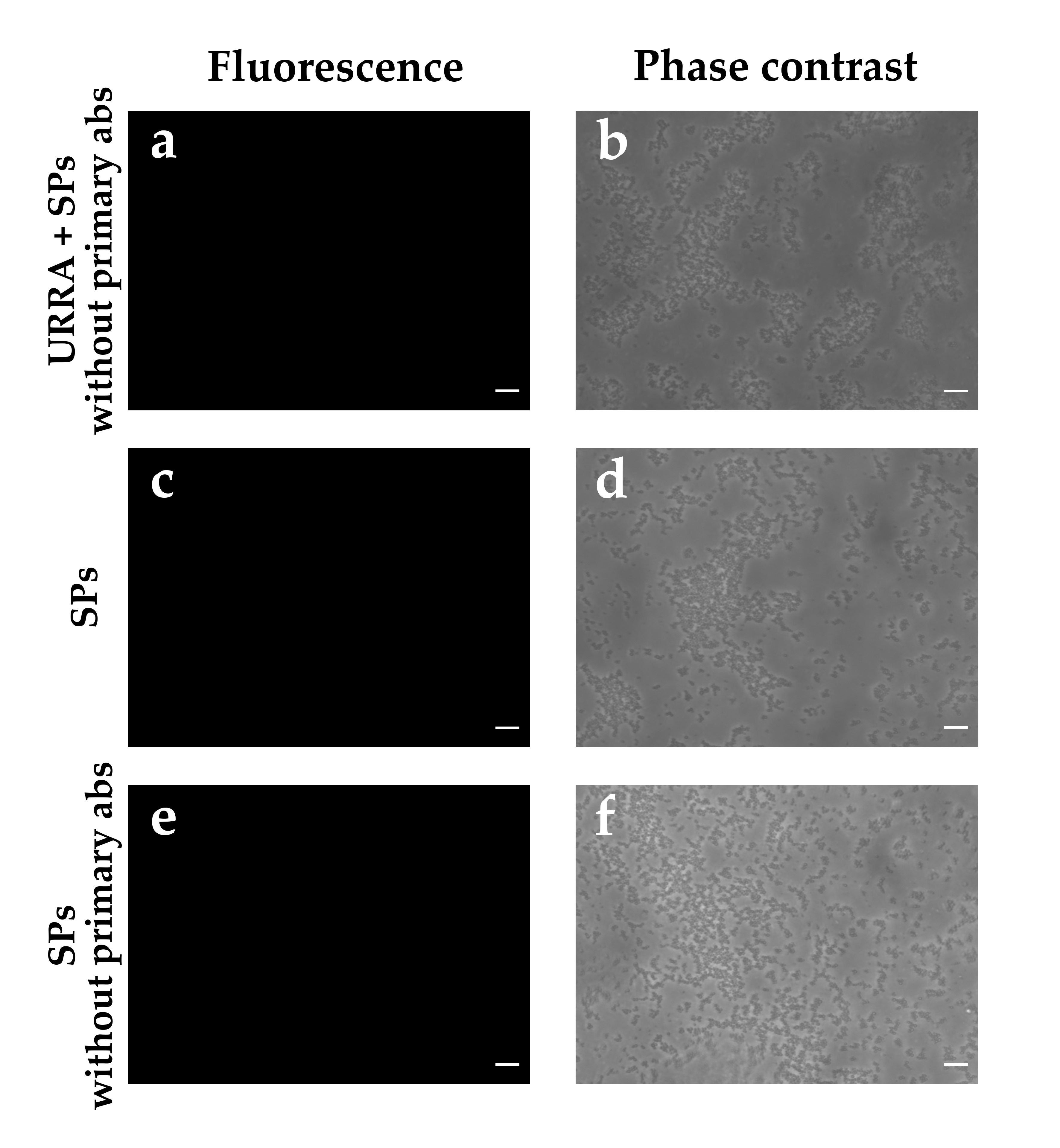

Supplement: Supplementary file 1 [file viruses-16-00438-s001.zip › Figure S1.tif]
